# A Rare Case of Erosive Esophagitis Due to Sarcina Ventriculi Infection

**DOI:** 10.7759/cureus.34230

**Published:** 2023-01-26

**Authors:** Christine Defilippo, Jana E DeJesus, Joseph M Gosnell, Suimin Qiu, Laurel Humphrey

**Affiliations:** 1 School of Medicine, University of Texas Medical Branch at Galveston, Galveston, USA; 2 General Surgery, University of Texas Medical Branch at Galveston, Galveston, USA; 3 Pathology, University of Texas Medical Branch at Galveston, Galveston, USA

**Keywords:** egd, esophagogastroduodenoscopy (egd), gerd, esophagitis, sarcinia ventriculi

## Abstract

*Sarcina ventriculi* is a Gram-positive anaerobic *coccus* found in soil that is a rare cause of inflammatory infections of the GI tract. This bacterium has a propensity for causing gastritis in patients with delayed gastric emptying. Of the 66 reported cases in the literature, 10 involved the esophagus. Symptoms of an esophageal infection are non-specific and may be mistaken for long-standing gastroesophageal reflux. We present a case of a 67-year-old female with chronic dysphagia and reflux diagnosed with erosive esophagitis caused by *Sarcina ventriculi*. Treatment strategies documented in the literature are reviewed.

## Introduction

*Sarcina ventriculi*, a Gram-positive anaerobic *coccus*, is a bacterium that thrives in acidic environments [1-3]. It is known for its characteristic packet-forming or tetrad morphology, which allows it to be identified on biopsy [3-5]. This bacterium is mostly found in the human stomach of those with delayed gastric emptying due to underlying conditions or the soil, which is thought to be the species’ natural habitat [4,6,7]. Infection due to *Sarcina ventriculi* is very rare and often leads to gastritis and, even more rarely, infection of the esophagus and small intestine [4,6]. Here we report a patient with a *Sarcina ventriculi* infection of both the esophagus and the duodenum. The patient presented with worsening of her long-standing gastroesophageal reflux and dysphagia. The diagnosis was made from a biopsy collected via esophagogastroduodenoscopy, and symptoms resolved after a two-week course of metronidazole.

## Case presentation

We present the case of a 67-year-old female with a past medical history significant only for long-standing gastroesophageal reflux who presented to the clinic with dysphagia and worsening reflux symptoms. She reported episodes of reflux every day after all meals. Her symptoms had been worsening over several months. She reported no abdominal pain, distention, nausea, vomiting, early satiety, or unintentional weight loss. Over-the-counter antacids provided her with some relief. She had previously taken pantoprazole for her symptoms. Her complete blood count (CBC) and basic metabolic panel (BMP) from her wellness exam two months prior were all within normal limits. Her last esophagogastroduodenoscopy (EGD) was 10 years ago and showed mild esophagitis. She was scheduled for a follow-up EGD.

**Figure 1:**
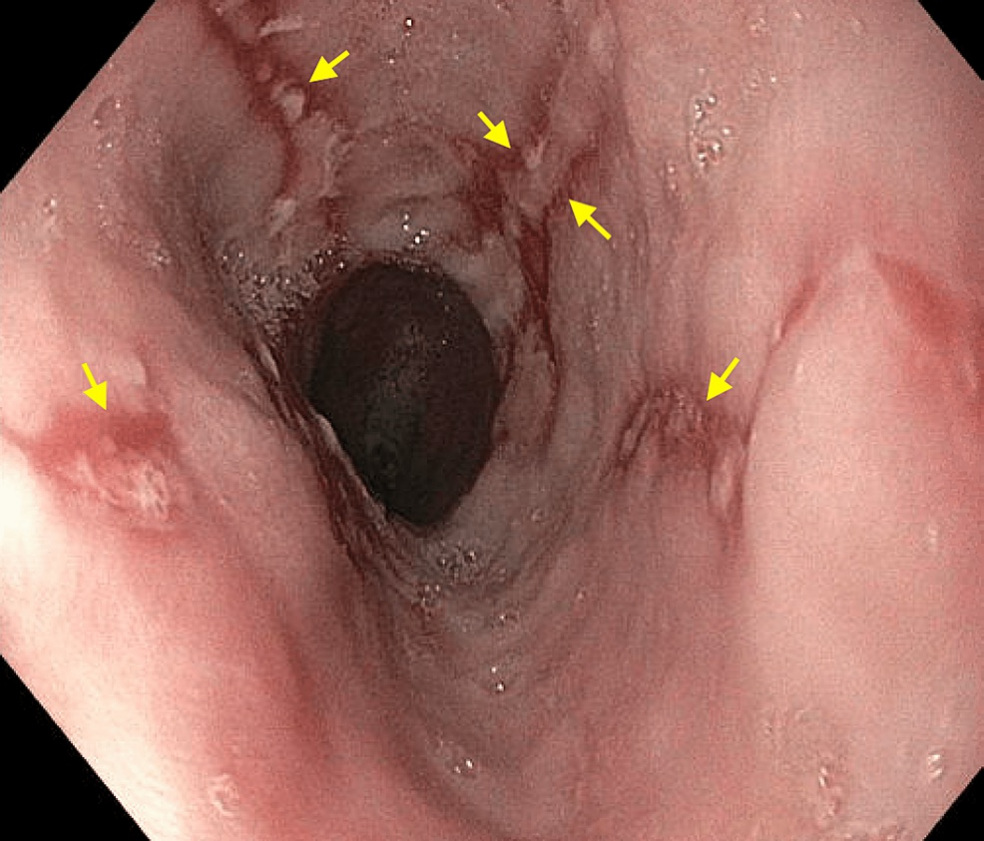
EGD findings at the lower third of the esophagus. Linear ulcerations (yellow arrows) with interspersed whitish plaques were identified.

**Figure 2:**
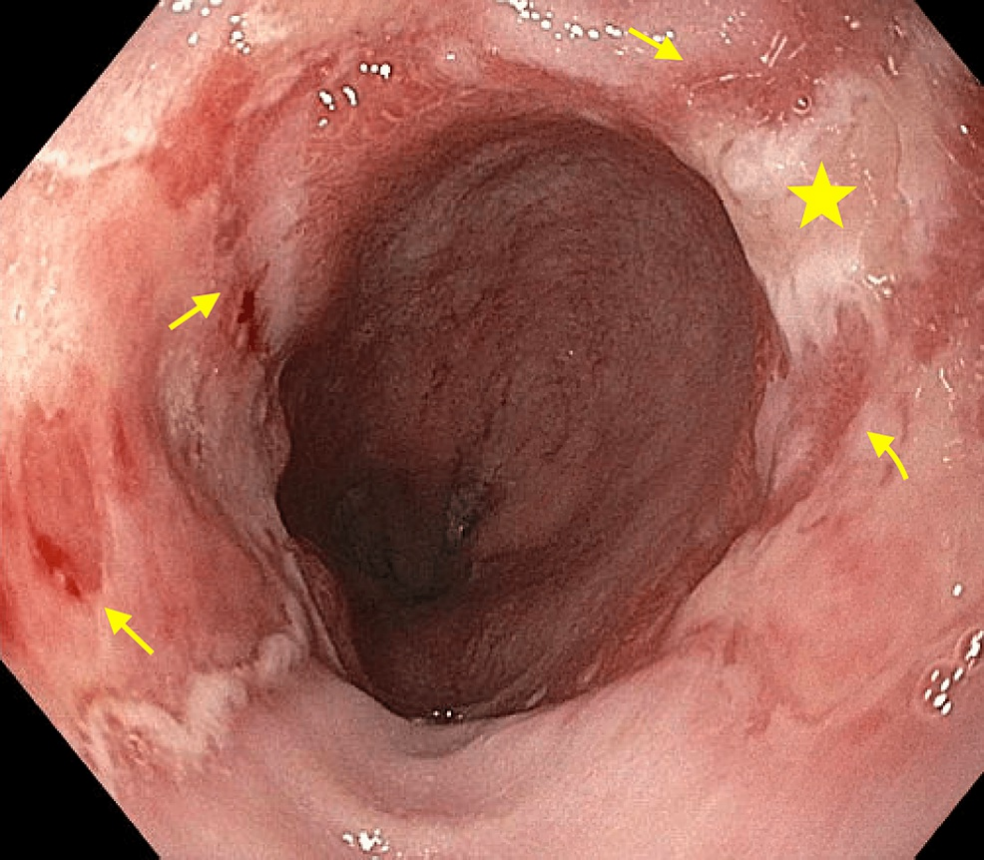
EGD findings at the gastroesophageal junction. Linear ulcerations (yellow arrows) and whitish plaques (yellow stars) continue through the gastroesophageal junction.

On the day of her EGD, diffuse esophagitis with linear ulcerations (LA Grade C) was seen from the middle third of the esophagus through the gastroesophageal junction. Whitish patches were interspersed between the ulcerations, resembling a candidal infection. Findings are illustrated in Figures 1, 2. Multiple biopsies were taken. The remainder of her EGD was grossly normal except for a few 2-4mm polyps at the gastric fundus. Routine biopsies at the gastric fundus, antrum, and first part of the duodenum were taken.

**Figure 3:**
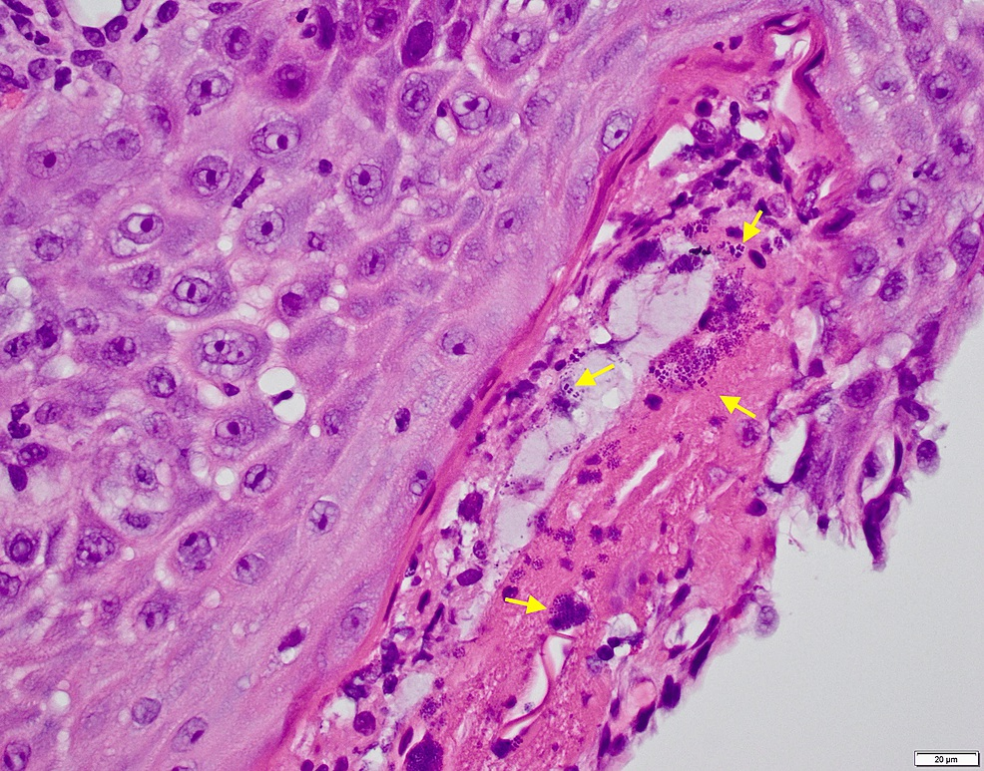
Distal esophageal biopsy showing features of erosive esophagitis and characteristic tetrad bacteria associated with acute inflammation. The slide is at 400X magnification. Yellow arrows point to tetrads of *Sarcina ventriculi*.

**Figure 4:**
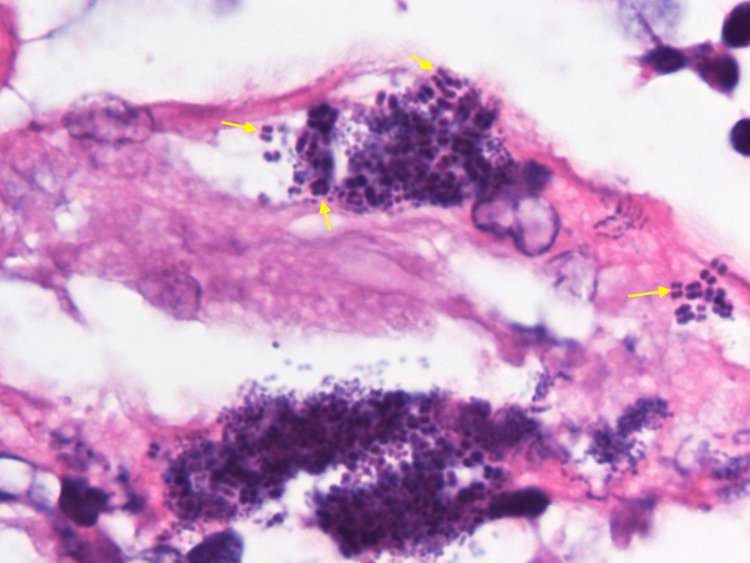
1000X magnification showing characteristic tetrad cocci of Sarcina ventriculi (yellow arrows).

The final pathology was negative for malignancy and *H. pylori*. Biopsies from the esophagus and duodenum were positive for Gram-positive *cocci* in tetrads consistent with *Sarcina ventriculi*. Histopathology is presented in Figure 3. The gastric biopsies were consistent with fundic gland polyps, likely due to previous proton pump inhibitor (PPI) use. The patient was started on a 14-day course of metronidazole and restarted on daily pantoprazole. She had near complete resolution of her symptoms at her two-week post-procedure visit, reporting reflux and dysphagia once per week. Figure 4 shows characteristic tetrad cocci of *Sarcina ventriculi*.

## Discussion

*Sarcina ventriculi* were first identified as a human pathogen in 1842 by John Goodsir after he saw a 19-year-old male who was vomiting acidic, fermented-smelling fluid every morning [1,2,8]. Unaware of what was causing this patient’s symptoms, Goodsir identified and named this micro-organism through microscopic investigation due to its highly characteristic and specific morphology. Charles Darwin notably contacted Goodsir after hearing of his discovery due to chronic stomach issues [1,6]. He had his vomitus sent to be evaluated by Goodsir to see if this newly discovered bacterium could be the cause.

Though initially identified in the 1840s, the first time it was isolated after culture was not until 1911 [2]. This confirmed that it was a living organism after many years of doubt. The morphological features of the bacterium delineated over time include cuboid shape, basophilic staining with hematoxylin-eosin stain, refractory, tetrad packet arrangement due to replication in two planes of growth, and packets that are the size of a red blood cell [2,3,5]. It is found most commonly in the soil and has been documented by the veterinary community as the cause of many gastrointestinal illnesses [1,2,4,7,9]. In humans, the most common location the bacterium inhabits is the stomach, followed by the esophagus and the duodenum. There is much debate as to whether the bacterium naturally colonizes the human intestinal tract or whether its presence is due to contamination from food intake [1,2,4,7].

The earliest clinical presentation identified in humans became known as ‘sarcinous vomiting’ due to its highly defined characteristic nature [2]. Patients exhibited obstinate vomiting of “acid, frothy, yeasty matters”. Over time the clinical presentation has been illustrated as abdominal pain, vomiting, nausea, delayed gastric emptying, dysphagia, abdominal distension, and weight loss [2]. However, patients may be asymptomatic, and diagnosis of this bacterium can be incidental [10,11]. Often, the bacterium is believed to be present due to prior clinical conditions, specifically due to pathologies that cause stasis of the gastric contents [6,12]. Conditions such as delayed gastric emptying, gastric outlet obstruction, and gastroparesis have the strongest associations [6,12]. These conditions provide context for the bacterium’s propensity for the stomach. Interestingly, the patient in this case report did not have any past medical history of type II diabetes. She additionally reported no symptoms of gastroparesis or gastric outlet obstruction. Cases where colonization is seen in the esophagus (15% of reported cases) and the duodenum (13% of reported cases) are rare [6,12]. The largest review of existing case reports by Marcelino documented the past medical history of each patient and the site of involvement [12]. In patients with esophageal involvement, a history gastroesophageal reflux was commonly reported [12]. Hiatal hernia and pathologies leading to chronic dysphagia may be additional contributors [12].

Infection of the esophagus or duodenum can lead to pathologies such as esophagitis or duodenal ulcers. These pathologies may progress to be life-threatening, such as in emphysematous gastritis, gastric perforation, and esophageal perforation [12]. Patients with a progression of the disease to this extent may present with hemodynamic instability and acute abdomen (i.e., diffuse abdominal pain and rigidity). Treatment would necessitate emergent surgical consultation and intervention.

Histological examination demonstrating Gram-positive *cocci* in tetrads is the only method of obtaining definitive diagnosis [1,4,6,12]. Reaction of the background mucosa has been found to vary from normal to active inflammation to metaplasia [13]. Endoscopy can exhibit findings that lead to a high clinical suspicion, such as ulceration, necrosis, or the presence of strictures, although these structural changes are not diagnostic of *S. Ventriculi* [6,12].

To rid the body of the bacteria, antibiotic regimens with Gram-positive and anaerobic coverage are used for 14 days [1,4,6,12]. The most commonly reported regimen in the literature is a combination of metronidazole and ciprofloxacin [4,12,14]. Other antibiotic regimens reported have included gentamicin, vancomycin, amoxicillin, fluconazole, amphotericin B, clarithromycin, and imipenem [12,14]. There is no consensus on the optimal regimen. The rarity of cases limits the data available for analysis. Even fewer cases with follow-up data are available. PPIs and prokinetic agents are added on a case-by-case basis. We recommended the addition of a PPI in this patient's case due to her underlying reflux and the extensive mucosal damage present.

Overall, the bacterium is very rare, with only 66 reported cases in the literature up to 2021 [12]. Most of the reported cases affected the stomach. Infections affecting the esophagus and duodenum are even rare. Little is known about the bacterium and its many characteristics. Increasing awareness of this entity amongst clinicians may increase the number of reported cases and aid in understanding the natural history of this disease and appropriate treatment regimens.

## Conclusions

The presence of *Sarcina ventriculi* in the human gastrointestinal tract is a rare occurrence. Approximately 15% of those affect the esophagus. It is often associated with conditions where gastric stasis or delayed gastric emptying is present and has the potential to lead to life-threatening complications. Antibiotics, including metronidazole, are the most common choice of treatment, although there has been no standard practice declared. With increasing identification and awareness of *Sarcina ventriculi*, hopefully more research and discoveries will be made, leading to concrete answers and a clear standard course of treatment/management.
